# Efficient Photocatalytic Degradation of Organic Pollutant in Wastewater by Electrospun Functionally Modified Polyacrylonitrile Nanofibers Membrane Anchoring TiO_2_ Nanostructured

**DOI:** 10.3390/membranes11100785

**Published:** 2021-10-14

**Authors:** Fahad A. AlAbduljabbar, Sajjad Haider, Fekri Abdulraqeb Ahmed Ali, Abdulaziz A. Alghyamah, Waheed A. Almasry, Raj Patel, Iqbal M. Mujtaba

**Affiliations:** 1 Department of Chemical Engineering, Faculty of Engineering & Informatics, University of Bradford, Bradford BD7 1DP, West Yorkshire, UK; thelastfahad@yahoo.com (F.A.A.); R.Patel@bradford.ac.uk (R.P.); 2 Department of Chemical Engineering, College of Engineering, King Saud University, P.O. Box 800, Riyadh 11421, Saudi Arabia; falhulidy@ksu.edu.sa (F.A.A.A.); aalghyamah@kus.edu.sa (A.A.A.); walmasry@ksu.edu.sa (W.A.A.)

**Keywords:** electrospinning, modified nanofibers, electrospray, TiO_2_/PAN composite, TiO_2_ coated PAN modified nanofibers, photocatalysis, band gap

## Abstract

In this study, polyacrylonitrile (PAN_P) nanofibers (NFs) were fabricated by electrospinning. The PAN_P NFs membrane was functionalized with diethylenetriamine to prepare a functionalized polyacrylonitrile (PAN_F) NFs membrane. TiO_2_ nanoparticles (NPs) synthesized in the laboratory were anchored to the surface of the PAN_F NFs membrane by electrospray to prepare a TiO_2_ NPs coated NFs membrane (PAN_Coa). A second TiO_2_/PAN_P composite membrane (PAN_Co) was prepared by embedding TiO_2_ NPs into the PAN_P NFs by electrospinning. The membranes were characterized by microscopic, spectroscopic and X-ray techniques. Scanning electron micrographs (SEM) revealed smooth morphologies for PAN_P and PAN_F NFs membranes and a dense cloud of TiO_2_ NPs on the surface of PAN_Coa NFs membrane. The attenuated total reflectance in the infrared (ATR-IR) proved the addition of the new amine functionality to the chemical structure of PAN. Transmission electron microscope images (TEM) revealed spherical TiO_2_ NPs with sizes between 18 and 32 nm. X-ray powder diffraction (XRD) patterns and energy dispersive X-ray spectroscopy (EDX) confirmed the existence of the anatase phase of TiO_2_. Surface profilometry da-ta showed increased surface roughness for the PAN_F and PAN_Coa NFs membranes. The adsorption-desorption isotherms and hysteresis loops for all NFs membranes followed the IV -isotherm and the H3 -hysteresis loop, corresponding to mesoporous and slit pores, respectively. The photocatalytic activities of PAN_Coa and PAN_Co NFs membranes against methyl orange dye degradation were evaluated and compared with those of bare TiO_2_ NPs.The higher photocatalytic activity of PAN_Coa membrane (92%, 20 ppm) compared to (PAN_Co) NFs membrane (41.64%, 20 ppm) and bare TiO_2_ (49.60%, 20 ppm) was attributed to the synergy between adsorption, lower band gap, high surface roughness and surface area.

## 1. Introduction

Hazardous industrial and agrochemical wastes left untreated pose an immediate threat to drinking water [1]. Therefore, to avoid water scarcity due to water pollution, the development of simple, efficient and affordable methods to remove water contaminants (such as dyes, phenols and pesticides) is crucial [2,3]. Photocatalysis, adsorption, filtration and sedimentation are some of the techniques used to remove biological and chemical pollutants from wastewater [3,4]. Photocatalysis has attracted a lot of interest from researchers as it is a simple, efficient, cost-effective and environmentally friendly process that allows complete degradation of various organic pollutants [5,6]. Moreover, photocatalysis is a sustainable method with strong oxidation capacity and lower energy consumption than other clean processes [7,8,9]. The most studied and active catalysts for the degradation of industrial and agrochemical wastes are titanium dioxide (TiO_2_) NPs, zinc oxide (ZnO) NPs and tungsten oxide (WO_3_) NPs. However, TiO_2_ NPs are widely used photocatalysts for environmental remediation: due to their chemical inertness, low cost, non-toxicity, photosensitivity and high oxidizing ability under ultraviolet (UV) light [2,10,11,12]. However, since photocatalysts are often in the form of particles, they tend to aggregate, which makes their removal after the photodegradation reaction a time-consuming and expensive process. In recent years, many articles have reported the combination of inorganic-organic hybrid membranes with good compatibility [13,14]. Studies also address the dispersion of photocatalysts on a large surface area membrane support material to improve their activity and recycling after use [15,16]. Polymer NFs membranes could be used for this purpose. The intensity of research on TiO_2_/NFs is increasing with each day. In the literature, there are very few research reports on this topic so far [17,18]. In some of the selected reports, the authors have used different strategies to demonstrate the feasibility of their studies. For example, Nasr et al. investigated the photocatalytic activity of BN–Ag/TiO_2_ composite NFs. They observed good photocatalytic activity for their composite, compared to pristine TiO_2_ NPs [19]. Zhuojun et al. prepared the network Au/TiO_2_ NFs for the photocatalysis of the rhodamine B (RB) dye. They observed a faster degradation of rhodamine B (RB) under UV-visible and natural light [20]. Jing et al. prepared mesoporous TiO_2_ electrospun NFs. They showed that the TiO_2_ anatase phase, prepared at 500°C, was very efficient against rhodamine B (RB) [21]. Studying the results published in the literature, it can be assumed that the combination of photolytically active ceramics and polymeric NFs offers polymer NFs will have advantages in both membrane filtration and photocatalysis of the industrial and agrochemical wastes. This strategy enables the development of hybrid NFs membranes with improved removal efficiency and selectivity, leading to a novel water treatment solution [22,23]. NFs membranes are prepared by various methods. These methods include drawing, phase separation, template synthesis and self-assembly [24]. However, electrospinning is considered as a flexible and successful technique to fabricate polymeric NFs with diameters ranging from nanometers to sub-micrometer [17,25]. Electrospinning is important compared to other methods because it is inexpensive, convenient to use, control of the process and environmental sustainability [26]. PAN: a well-known polymer with excellent stability, environmental friendliness, economic profitability and easy spinnability and functionalization has recently received much attention compared to other polymers [27]. This is because PAN is a versatile polymer: it can be used to proudce ultrafiltration membranes, hollow fibers for reverse osmosis, fibers for textiles and oxidized PAN fibers. Electrospun PAN NFs can serve as a potential support for catalytic materials. Moreover, since electrospun PAN-based NFs have low density and are flexible, they can easily float on a liquid or fixed at the desired location in reactors [28,29].

The present work aims to develop a novel membrane for the treatment of dye-contaminated wastewater. Two approaches were followed in this study. First, a PAN_P NFs membrane was prepared by electrospinning. The PAN_P NFs membrane was functionalized to a PAN_F NFs membrane. The PAN_F NFs membrane was then electrospray with TiO_2_ NPs to produce a PAN_Coa NFs membrane. Second, TiO_2_ NPs were embedded in the fibers during electrospinning to produce a PAN_Co NFs membrane. The PAN_Coa and PAN_Co NFs membranes were used to study their photocatalysis behavior toward methyl orange. To the best of our knowledge, no report of a similar study has been published in the literature.

## 2. Materials and Methods

### 2.1. Materials

PAN (average molecular weight (Mw) 150,000), dimethylformamide (DMF(C_3_H_7_NO)), diethylenetriamine (DETA (C_4_H_13_N_3_)), methyl orange ((MO) C_14_H_14_N_3_NaO_3_S), Titanium (IV)-n-butoxide (Ti(C_4_H_9_O)_4_), glacial acetic acid (CH_3_COOH), sulfuric acid (H_2_SO_4_) and sodium hydroxide (NaOH) were supplied by Sigma-Aldrich (St. Louis, MO, USA). Sodium carbonate anhydrous (Na_2_CO_3_) was purchased from Paneac Quimica S.L.U. (Barcelona, Spain). *NANON*-01A electrospinning machine (MECC, Fukuoka, Japan) was used to prepare NFs membranes and electrospray TiO_2_ NPs onto the surface of the PAN_F NFs membrane. Distilled water was used as a solvent to prepare dye solutions. All the chemicals were of analytical grade and were used without further purification. Locally prepared Teflon frames were used to fix the edges of the membrane during treatment.

### 2.2. Preparation of the PAN_P NFs Membrane

A 10 wt% PAN solution was prepared by dissolving 1 g PAN powder in 10 mL DMF. The solution was agitated for 24 h at room temperature on a magnetic stirrer. The resulting homogeneous PAN solution was transferred to a 5 mL plastic syringe. The plastic syringe was placed in a controlled flow pump. PAN_P NFs membrane was prepared by electrospinning at optimized conditions (distance between needle and collector 150 mm, needle diameter 0.8 mm, applied voltage 20 kV, solution flow rate 0.8 mL/h and the cylindrical collector speed 100 revolutions per minute (rpm). The NFs membrane was peeled off the aluminum foil and dried in a vacuum oven at 50 °C and −0.1 MPa before being stored for grafting. The thickness of the membrane was ~0.123 ± 0.5 mm.

### 2.3. Preparation of PAN_F NFs Membrane

PAN_F NFs membrane was synthesized by immersing PAN_P NFs membrane in a 250-mL beaker containing 10 mL DETA solution (2.3 M in ethanol) and 100 mL sodium carbonate (0.83 g in distilled water). The mixture was agitated on a water bath at 90 °C for 5 h. After completion of the reaction, the PAN_F NFs membrane was washed several times with distilled water. After washing, the NFs membrane was dried in an oven at 60 °C until constant weight and stored for characterization. The conversion of the nitrile group to DETA was calculated as follows.

(1) $$C_{n}=\frac{W_{1}-W_{0}}{W_{0}}\;\frac{M_{0}}{M_{1}}\times 100$$

where C_n_ is the % of the nitrile group in PAN that is converted to the amine group, W_0_ is the weight of PAN_P NFs membrane before the reaction, W_1_ is the weight of PAN_F NFs membrane after the reaction. M_1_ is the MW of DETA (103.17 g/mol), and M_0_ is the MW of acrylonitrile monomer (53.06 g/mol).

### 2.4. Synthesis of TiO_2_

TiO_2_ NPs were synthesized by the chemical precipitation method. Titanium (IV)-n-butoxide (Ti (OBu) 4) (26 mL), ethanol (53 mL) and glacial acetic acid (43 mL) were added to a reaction container. The mixture was stirred at 55 °C for 1 h. After mixing, 4 mL of sulfuric acid was added dropwise to the mixture using a dropper. The reaction was stopped after gel formation. The residual solvent in the gel was extracted by centrifuging the mixture at 3500 rpm. The gel was dried at 100 °C for 2 h before calcination at 600 °C for 6 h to produce the nanocrystalline anatase TiO_2_ NPs.

### 2.5. Preparation of PAN_Coa NFs Membrane

**Scheme 1:**
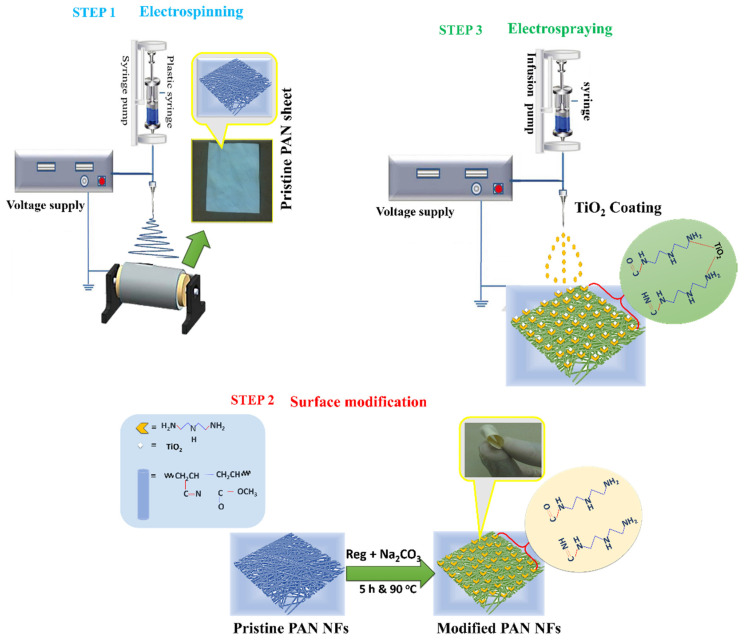
Preparation of PAN_P NFs membrane by electrospinning (**Step 1**), synthesis of PAN_F NFs membrane (**Step 2**), coating of PAN_F NFs membrane with TiO_2_ by electrospray (**Step 3**).

The suspension of 2 wt% TiO_2_ NPs was prepared by dispersing 0.2 mg TiO_2_ NPs in 10 mL DMF. The suspension was ultrasonicated (VCX500, Sonics and Material, Inc., Newtown, CT, USA) for 10 min. The suspension was added to a 20 mL plastic syringe. The syringe was placed in a controlled flow pump. The suspension was then electrosprayed onto the surface of the PAN_F NFs membrane at an applied voltage of 20 kV. The flow rate of the suspension was 0.8 mL/h. The distance between the collector and the needle tip was 150 mm. The schematic (Scheme 1, Step 3) shows the prepraration, synthesis and coating of the PAN_Coa NFs membrane.

### 2.6. Preparation of PAN_Co NFs Membrane

**Scheme 2:**
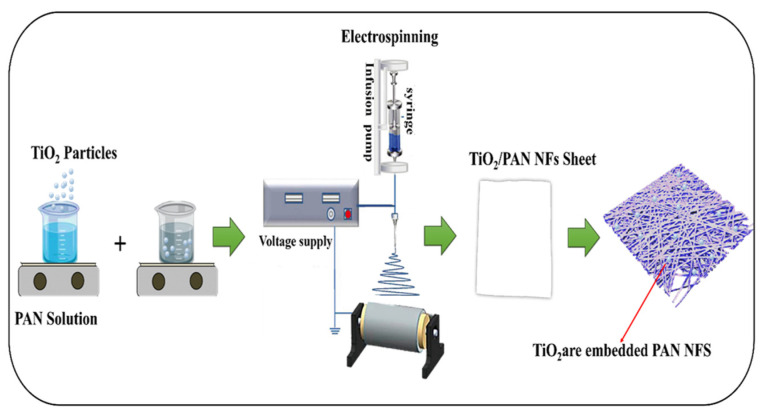
Preparation of the TiO_2_/PAN solution and its electrospinning to fabricate PAN_Co NFs membrane.

TiO_2_/PAN composite solutions were prepared by adding different wt% TiO_2_ NPs (1, 2, 4 wt%) to 10 wt% PAN solutions. Each solution was Ultra sonicated (VCX500, Sonics and Material, INC., Newtown, CT, USA) for 10 min to make the dispersion of TiO_2_ NPs homogeneous in the PAN solution. Each solution was added to a 20 mL plastic syringe. The syringe was placed in the controlled flow pump. Electrospinning of each solution was carried out at previously optimized conditions (applied voltage 20 kV, solution flow rate 0.8 mL/h and distance between collector and needle tip 150 mm) The scheme (Scheme 2) shows the preparation of the composite membrane by electrospinning.

### 2.7. Characterization

The surface morphologies of PAN_P, PAN_F, PAN_Coa and PAN_Co NFs membranes were studied using an SEM (JSM-2100F, Jeol, Tokyo, Japan). The membrane samples for the SEM study were attached to holders with carbon tape. The holders were placed in the platinum sputtering machine and the samples were coated with platinum. The surface morphology of the platinum-coated membrane samples was then examined using SEM under high vacuum. An ATR spectrometer (Nicolet iN10 FTIR microscope with a germanium microtip, Thermo Scientific, Winsford, UK,) was used to study the ART-FTIR spectra of PAN_P, PAN_F, PAN_Coa and PAN_ Co NFs membranes in the spectral range 900–2300 cm^−1^. TEM (JEM-2100F, Jeol, Tokyo, Japan) images were also used to examine the shape, size and crystallinity of the green-produced TiO_2_ NPs. The EDX of the TiO_2_ NPs was also taken using the EDX available with the TEM. The surface area, pore size, pore-volume and total area in the pores of PAN_P, PAN_F, PAN_Coa and PAN_Co NFs membranes were determined using Micromeritics (Gemini VII, 2390 Surface Area and Porosity, Norcross, GA, USA). Prior to analysis, the sample was degassed at 150 °C for 120 min under N_2_ flow to remove moisture. The N_2_ adsorption-desorption isotherm was examined at STP in the relative pressure range of 0.0 to 0.1. XRD data were recorded at 40 kV and 30 mA with Cu Kα radiation (1.540 Å). The XRD diffractograms were recorded in the 2θ° range from 20° to 80° (Bruker AXS D8 Advance XRD, Billerica, MA, USA). Surface roughness was determined using a contour GT-K 3D optical microscope (Bruker®, Billerica, MA, USA), non-contact 3D surface metrology and interferometry. Vertical scanning interferometry was used to measure the samples, with a 5× Michelson magnification lens, a field of view of 1.0 × 1.0 mm^2^, a Gaussian regression filter, a scan speed of 1× and a threshold of 4. The samples were placed on the stage and manually adjusted to obtain an image on the screen. The microscope is controlled by Vision 64 software (Bruker®, Billerica, MA, USA), which handles instrument settings, data analysis and graphical output. Vertical scanning interferometry, which uses a broadband (usually white) light source, was used for the measurement. It is efficient for measuring objects with rough surfaces as well as objects with adjacent pixel-height differences greater than 135 nm. Each sample was scanned at three different locations with three different intervals and then averaged to obtain the roughness value (Ra).

### 2.8. Degradation of Methyl Orange

PAN_Coa and PAN_Co NFs membranes and bare TiO NPs were used to degrade methyl orange dye. Different weights PAN_Coa (20, 40, 60 mg) of the membranes were added separately to 100 mL of the 10, 20 and 30 ppm methyl orange dye solutions. From the results, the optimum dose of membrane photocatalysts and the concentration of the dye were determined. 60 mg PAN_Co (containing 1, 2 and 4 wt% TiO_2_ NPs) membranes and bare TiO_2_ NPs were added separately to a photocatalyst reactor containing 20 ppm methyl orange solution. The catalytic material and methyl orange were stirred in the dark for 1 h to establish adsorption-desorption equilibrium. During the photocatalytic reaction, an incandescent bulb (450 W) served as the light source. The concentration of methyl orange in the solution was determined every 30 min using a UV/visible spectrophotometer. The kinetics of the photocatalytic oxidation of the methyl orange on PAN_Coa and PAN_Co NFs membranes followed pseudo-first-order kinetics (Equation (2)). The degradation of methyl orange is calculated according to Equation (3) [23].

(2) $$\ln\left(\frac{A_{0}}{A_{t}}\right)=kK=K_{app}t$$

(3) $$\mathrm{Degradation}=1-\frac{A_{t}}{A_{0}}$$

where *A*_0_ is the initial UV/visible spectrum of methyl orange, *A_t_* is the UV/visible spectrum of methyl orange at illumination time *t*, *k* is the reaction rate constant and *K* is the absorption coefficient of the reactant. *A* plot of ln(*A*_0_/*A_t_*) versus time represents a straight line whose slope corresponds to the apparent first-order rate constant *K_app_* [30].

## 3. Results and Discussion

### 3.1. SEM Analysis

**Figure 1:**
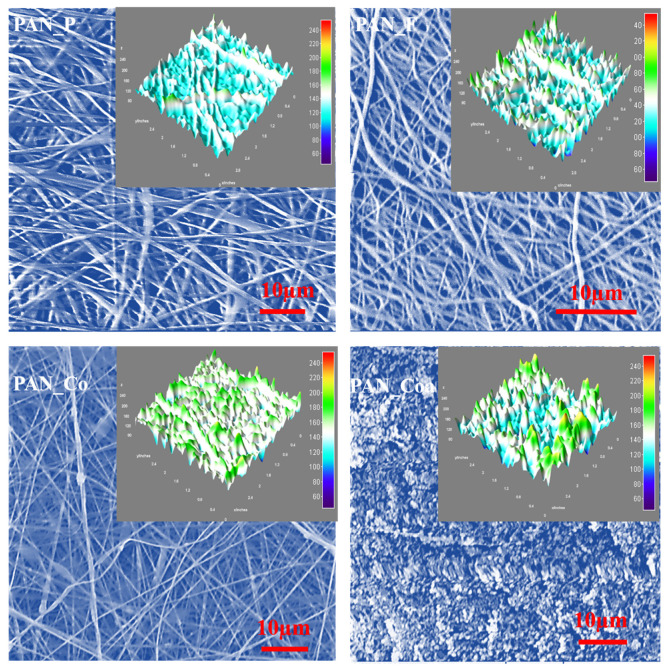
SEM micrographs of the PAN_P, PAN_F, PAN_Co and PAN_Coa NFs membranes. The 3D surface plots of the same are shown in the inset.

Figure 1 shows SEM micrographs of PAN_P, PAN_F, PAN_Coa and PAN_ Co NFs membranes. The PAN_P NFs membrane had a smooth and uniform morphology. Similarly, no cracks or degradation of NFs were observed in the PAN_F NFs membrane. The consistent physical texture of the membranes is an indication that the fibers resisted morphological deformation during chemical treatment. The conversion of nitrile to DETA calculated using Equation (1) was ~50% [31]. Embedding TiO_2_ NPs in NFs (PAN_Co) during electrospinning without changing the spinning conditions resulted in elongated beaded fibers that appeared as dark clouds in the SEM micrographs. The appearance of these dark clouds is attributed to an increase in PAN solution viscosity due to the addition of TiO_2_ NPs. TiO_2_ NPs in a polymer matrix lead to physical crosslinking interactions between the polymer molecules. The Ti-O bond in TiO_2_ is polar. Hydrogen bonding between the oxygen of TiO_2_ and the polar hydrogen of the polymer molecules has been reported in the literature [32]. Crosslinking increases the viscosity of the solution. The increase in solution viscosity beyond a threshold value not only hinders the flow of solution through the needle tip, but also reduces the evaporation of the solvent during the flight of fibers between the needle tip and the collector. These two factors lead to the formation of beaded fibers [33]. Electrospraying of TiO_2_ NPs onto the surface of PAN_F membrane resulted in dense clouds of TiO_2_ NPs on the membrane [34]. The 3D surface plots, which can be seen in the inset of the micrographs show a significant change in surface roughness. The measurements and the values of the surface roughness of the membranes are discussed in the surface roughness section.

### 3.2. TiO_2_ NPs Morphology and Phase

**Figure 2:**
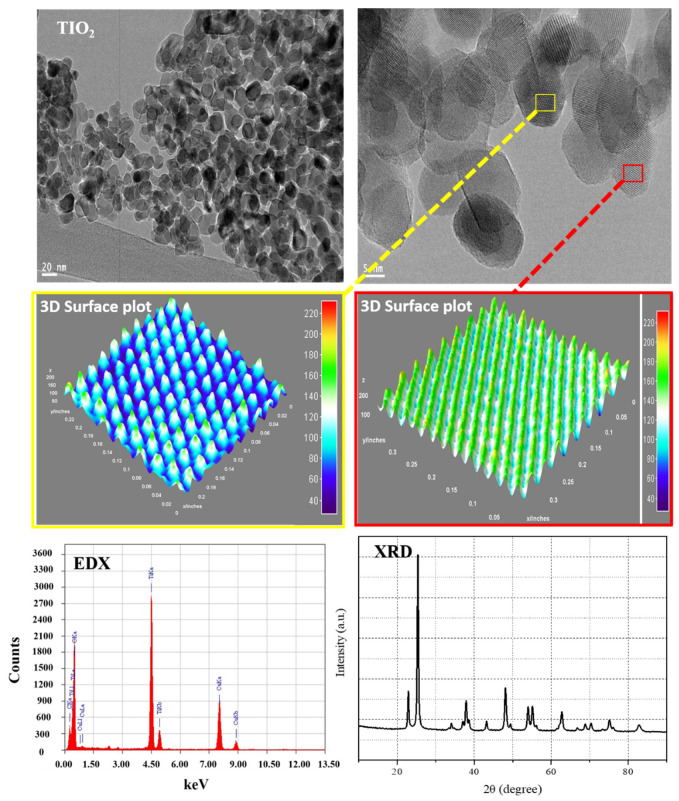
TEM, 3D surface plot, EDX and XRD of the synthesized TiO_2_ NPs.

The TiO_2_ NPs synthesized by the homogeneous precipitation method at 600 °C were characterized by TEM, EDX and XRD (Figure 2). TEM images showed that the average particle size varied between 18 and 35 nm. Moreover, the particles were spherical and of good crystallinity. The HRTEM images showed well-resolved lattice features and distinct lattice fringes with no structural deviations from the anatase phase. The spacing of ~0.35 nm between adjacent lattice fringes of the anatase plane (101), confirms the XRD results discussed below [3]. The 3D surface plots of TiO_2_ NPs were prepared from the HRTEM images and highlighted with green-blue color lines. Examination of the 3D surface plots revealed that calcination had no effect on the long range atomic organization. Therefore, the surfaces appeared atomically flat. EDX spectroscopy for elemental analysis performed using EDX coupled with TEM, confirmed "Ti" as the major element. The XRD diffraction pattern confirmed the anatase phase of the TiO_2_ NPs. The diffraction pattern of anatase phase was found in crystal planes (101), (004), (200), (105), (211), (204), (116), (220), (215) and (303) crystal planes (JCPDS-21-1272). The anatase phase of TiO_2_ NPs reported in the literature has been shown to be photoactive and useful for wastewater treatment and purification [35,36].

### 3.3. ATR-FITR Study

**Figure 3:**
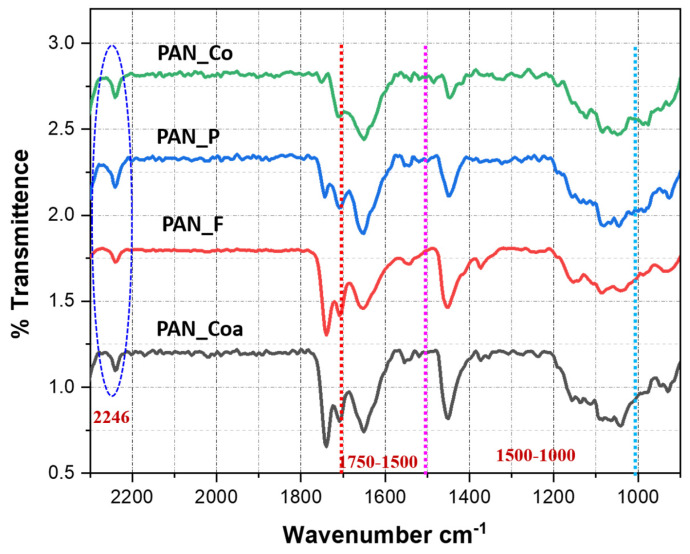
ATR-IR spectra of the PAN_P, PAN-F, PAN-Co and PAN_Coa NFs membranes.

The ATR-IR spectra of PAN_P, PAN_F, PAN_Coa and PAN_Co NFs NFs membranes are shown in Figure 3. The spectrum of PAN_P NFs showed bands at 1739 cm^−1^ (C=O stretching), 1000–1475 cm^−1^ (C–N and C–H stretching) and 2246 cm^−1^; –C≡N stretching, i.e., acrylonitrile unit [37,38]. During the synthesis of PAN_F NFs membrane, the intensity of the band decreased at 2246 cm^−1^. The decrease in the intensity of the band at 2246 cm^−1^ during the synthesis of PAN_F NFs membrane indicates that –C≡N was the reaction site. The band at 1596 cm^−1^ in the spectrum of PAN_F NFs membrane, which increased in intensity, was assigned to the N-H in the amine group [39,40,41]. The decrease in band intensity at 2246 cm^−1^ and the increase in band intensity at 1596 cm^−1^ in the spectrum of PAN_F NFs membrane both show the conversion of the –C≡N group to an amine group. The bands for PAN_Coa and PAN_Co NFs NFs membrane showed no change in their positions and intensities.

### 3.4. XRD of Membranes

**Figure 4:**
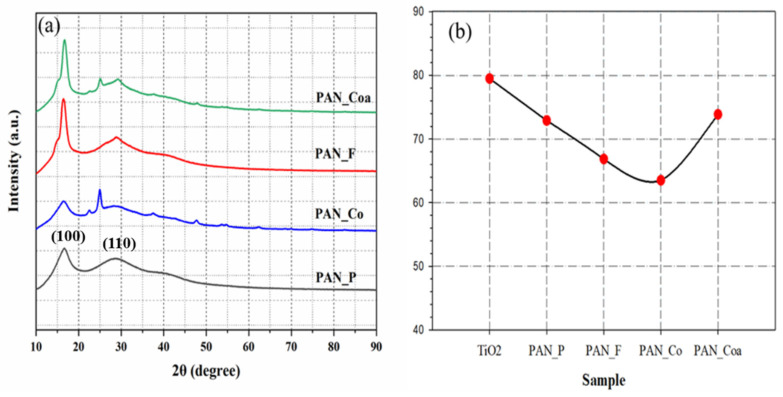
(**a**) XRD plots of PAN_P, PAN_F, PAN_Co and PAN_Coa NFs membranes and (**b**)% crystallinity calculated with Origin 18 software from the XRD plots in Figure 2 and Figure 4.

XRD was used to study the diffraction patterns and crystallinity of PAN_P, PAN_F, PAN_Coa and PAN_ Co NFs membranes (Figure 4a). The XRD diffraction patterns of PAN_P, PAN_F, PAN_Co and PAN_Coa NFs membranes showed a broad amorphous halo at 2θ ≈ 30° and a crystalline peak at 2θ ≈ 17°corresponding to the (110) and (100) crystalline planes [41,42] The diffraction patterns of PAN_Co and PAN_Coa NFs membranes also showed the diffraction pattern for the anatase phase of TiO_2_. The diffraction pattern of TiO_2_ NPs in the anatase phase is explained in Section 3.2. The appearance of the diffraction pattern of TiO_2_ NPs in the anatase phase in the diffraction patterns of PAN_Co and PAN_Coa NFs membranes confirms the presence of TiO_2_ in these. The % crystallinity of TiO_2_ NPs, PAN_P, PAN_F, PAN_Co and PAN-Coa NFs membrane was calculated using Origin 18 software and Equation (4) (Figure 4b). The % crystallinity showed a variation in the crystallinity of these samples. The crystallinity of TiO_2_ was 79.50%. A similar crystallinity for the anatase phase of TiO_2_ NPs is also reported in the literature [39,40,41,42,43].

Crystallinity (%) = Acrystal/Atotal × 100(4)

PAN_P NFs membrane had a slightly higher % crystallinity than PAN_F and PAN_Co NFs membranes. The higher % crystallinity of PAN_P NFs membrane was attributed to the robust arrangement of polymer chains and their packing into a crystalline structure. PAN_F and PAN_Co NFs membranes showed a significant decrease in % crystallinities. The decrease in % crystallinity of the PAN_F NFs membrane is attributed to the disruption of polymer chain packing due to the incorporation of amine functionality into the chemical structure of PAN. A similar effect can also be attributed to the decrease in the % crystallinity of PAN_Co NFs membrane due to the addition of TiO_2_ NP [32,44,45]. In addition to the above reasons, we can also conclude that the crystallization in PAN_F and PAN_Co NFs membranes was inhibited by varying the solvent evaporation and polymer solidification [42]. The % crystallinities of the PAN_Coa and PAN_P NFs membranes were comparable. The significant improvement in the % crystallinity for PAN_Coa NFs membrane can be attributed to the balancing effect of the crystallinities of PAN_F NFs membranes and the coated TiO_2_ NPs.

### 3.5. Surface Roughness

**Figure 5:**
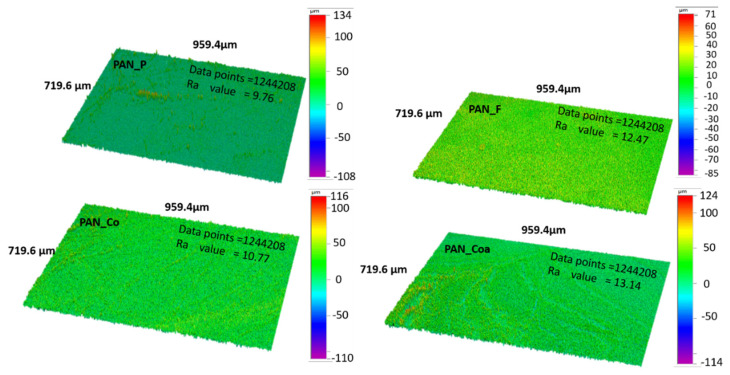
Surface roughness of the PAN_P, PAN_F, PAN_Coa and PAN_Co NFs membranes.

The surface profilometry results for PAN_P, PAN_F, PAN_Co and PAN_Coa NFs membranes are shown in Figure 5. The roughness of the membrane surfaces varied as a function of treatment and composition. PAN_P NFs membrane had the lowest surface roughness, while PAN_Coa NFs membrane had the highest, followed by PAN_F and PAN-Co NFs membranes. The highest surface roughness of PAN_Coa NFs membranes is attributed to the synergy of chemical treatment and random deposition of TiO_2_ NPs on the membrane surface. Similar results indicating increased surface roughness of the membrane due to the chemical modification of the membrane and the random deposition of TiO_2_ NPs on the membrane surface have been reported in the literature [46,47]. The increased surface roughness of the PAN_F and PAN_Co NFs membranes compared to the PAN_P NFs membrane could be explained by the chemical modification of the membrane ((PAN_F) as mentioned earlier) and the random dispersion of TiO_2_ NPs in the polymer matrix(PAN_Co) [32].

### 3.6. Porosity and Surface Areas

**Figure 6:**
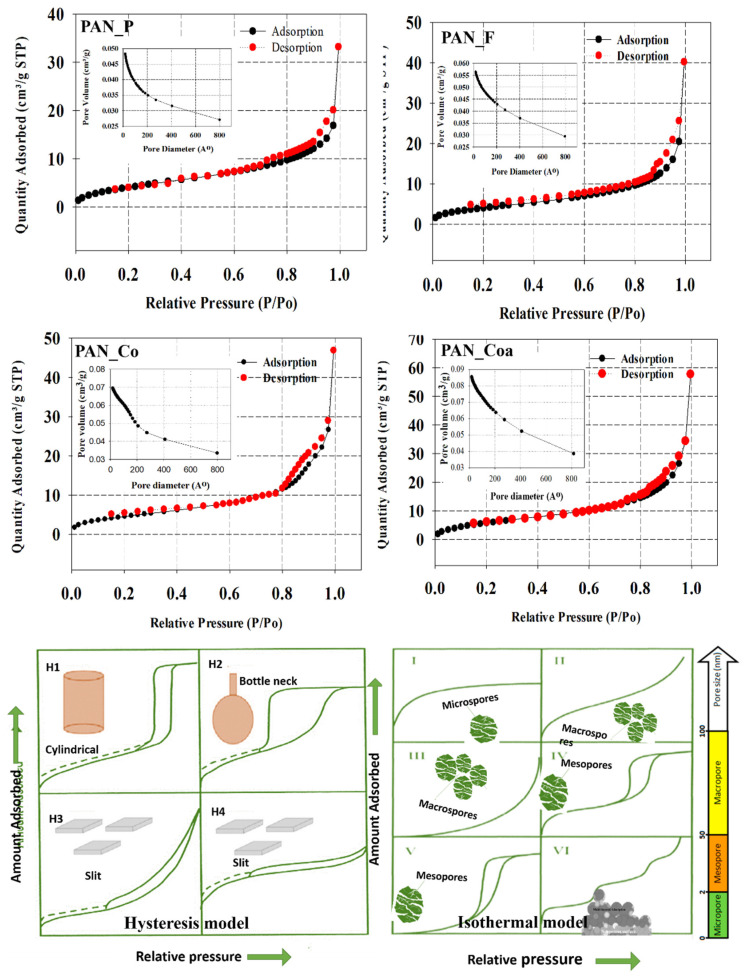
Adsorption-desorption isotherms of the PAN_P, PAN_F, PAN_Co and PAN_Cao NFs membranes under nitrogen atmosphere. IUPAC classified hysteresis and isotherm models.

BET was used to evaluate the surface area and porosity of PAN_P, PAN_F, PAN_Co and PAN_Coa NFs membranes using nitrogen gas as the adsorbate. BET is the most commonly used technique to evaluate porous materials with meso(diameter range 2–50 nm) and micro (diameter 2 nm) pore dimensions. Figure 6 shows the adsorption-desorption isotherms and hysteresis for PAN_P, PAN_F, PAN_Co and PAN_Coa NFs membranes. The porosity of a material is defined: as the ratio of the volume of pores and voids to the total volume of the material. The International Union of Pure and Applied Chemistry (IUPAC) convention is a standard used to classify isotherms and hysteresis to represent the associated different pore sizes and pore channels in materials There are six types of isotherms described in the literature that characterize the porosity of materials. These are type I (microporous), II, III and VI (non-porous or macroporous), and IV and V (mesoporous) (Figure 6) [48,49,50]. Similarly, IUPAC has also divided hysteresis into four types. Hysteresis characterizes the pore channels in materials. These are H1 (cylindrical- pore channels or agglomerates of generally homogeneous spheres), H2 ( bottleneck-type pores or constrictions), H3 (slit pores) and H4 (smaller slit pores)(Figure 6) [51]. The adsorption-desorption isotherms and hysteresis for PAN_P, PAN_F, PAN_Co and PAN_Coa NFs membranes were identical and classified as type IV(mesopores) and H3 (slit-like pores). Similar results for the pores of NFs membranes have been published in the literature [52,53]. The surface area of the PAN_F NFs membrane showed no significant change by chemical treatment. However, the pore volume, pore size and total area in the pore increased (Table 1). These changes in the PAN_F NFs membrane are attributed to the relaxation of polymer chains after chemical treatment. The surface area, the pore volume, pore size and total area in the pore for PAN_Co, and PAN_Coa NFs membranes increased (Table 1). This increase for PAN_Co NFs membrane could be attributed to the addition of the TiO_2_ NPs and for PAN_Coa NFs membrane could be attributed to the synergy of the chemical treatment and TiO_2_ NPs coating.

### 3.7. Photocatalytic Study onto Synthesized Membranes

TiO_2_: a semiconductor material that has recently been the subject of intense study because of its low cost, photocatalytic activity, biocompatibility, nontoxicity and good stability. It occurs in a variety of forms. These include rutile, brookite and anatase. The band gap energy of rutile TiO_2_ is 3.03 eV and anatase is 3.2 eV. These band gaps correspond to a wavelength in the near UV range (380–387 nm). Thus, they can be excited in the UV range.

**Figure 7:**
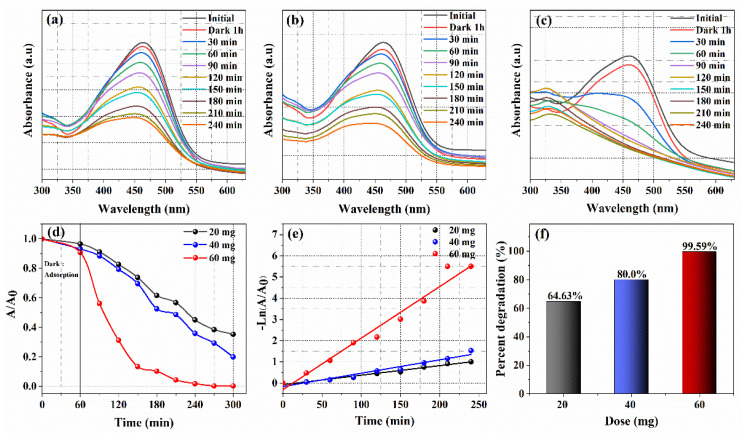
Dose study of photocatalytic PAN_Coa NFs membrane. Spectrophotometer spectra at different time intervals and photocatalytic doses (**a**) 20 mg, (**b**) 40 mg and (**c**) 60 mg, (**d**,**f**) degradation and (**e**) kinetics The concentration of methyl orange was constant (10 ppm).

**Figure 8:**
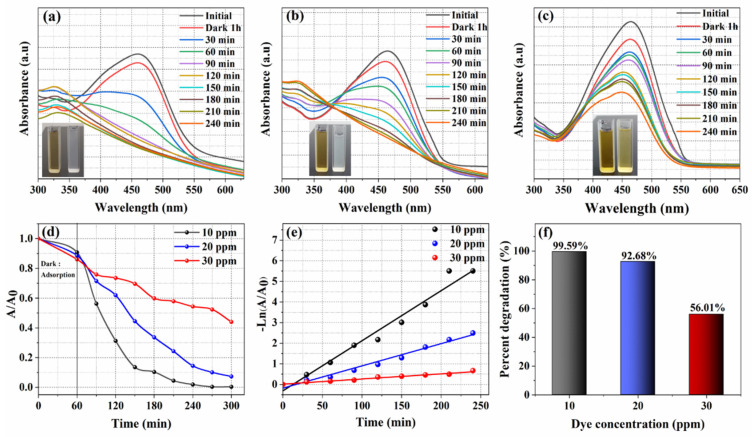
Concentration study of methyl orange. Spectrophotometer spectra at different time intervals and methyl orange concentrations (**a**) 10 ppm, (**b**) 20 ppm and (**c**) 30 ppm. The dose of the PAN_Coa NFs membrane was kept constant (60 mg), (**d**,**f**) degradation and (**e**) kinetics.

It is already known that the values of the band gap are influenced by the synthesis process, the doping of the crystalline network with other materials (ceramic or carbon) and the crystal size of the semiconductor. However, there are very few studies on the combination of ceramic particles with polymers for photocatalysis. Coating the surface of NFs membrane with TiO_2_ NPs by electrospray has hardly been explored [54]. Figure 7 shows the photocatalytic abilities of the PAN_Coa NFs membrane to degrade a methyl orange dye solution using a UV light source. Blank experiments conducted with the PAN_P NFs membrane resulted in negligible dye degradation. However, studies conducted with the PAN_Coa NFs membrane and in the absence of a light source showed slight adsorption of the dye on the NFs membrane. The adsorption was attributed to the presence of the amine group in the membrane. The optimization of the dosage of PAN_Coa NFs membrane is shown in Figure 7a–f. Figure 8a–f shows the optimization of the concentration of methyl orange. As shown in Figure 7a–f, increasing the dose of PAN_Coa NFs membrane from 20 mg to 60 mg resulted in maximum dye degradation (10 ppm, 99.59% (Figure 7f). Using 60 mg dose and changing methyl orange concentration from 10 ppm to 30 ppm (Figure 8a–f) showed that increasing the concentration decreased the photocatalyst membrane activity (Figure 8f). The optimum methyl orange concentration with maximum photocatalyst activity was 20 ppm. Therefore, 60 mg dosage and 20 ppm were selected as the optimum values for further experiments.

**Figure 9:**
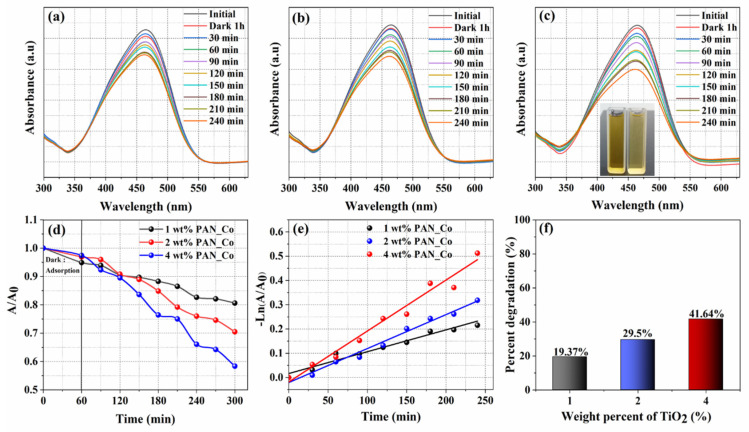
Photocatalytic activity of PAN_Co NFs membrane with different wt% of TiO_2_ NPs. Spectrophotometer spectra at different time intervals and wt% of TiO_2_ (**a**) 1 wt%, (**b**) 2 wt% and (**c**) 4 wt%. (**d**,**f**) degradation and (**e**) kinetics. The dose of photocatalytic membrane (60 mg) and concentration of the dye (20 ppm) were kept constant.

**Figure 10:**
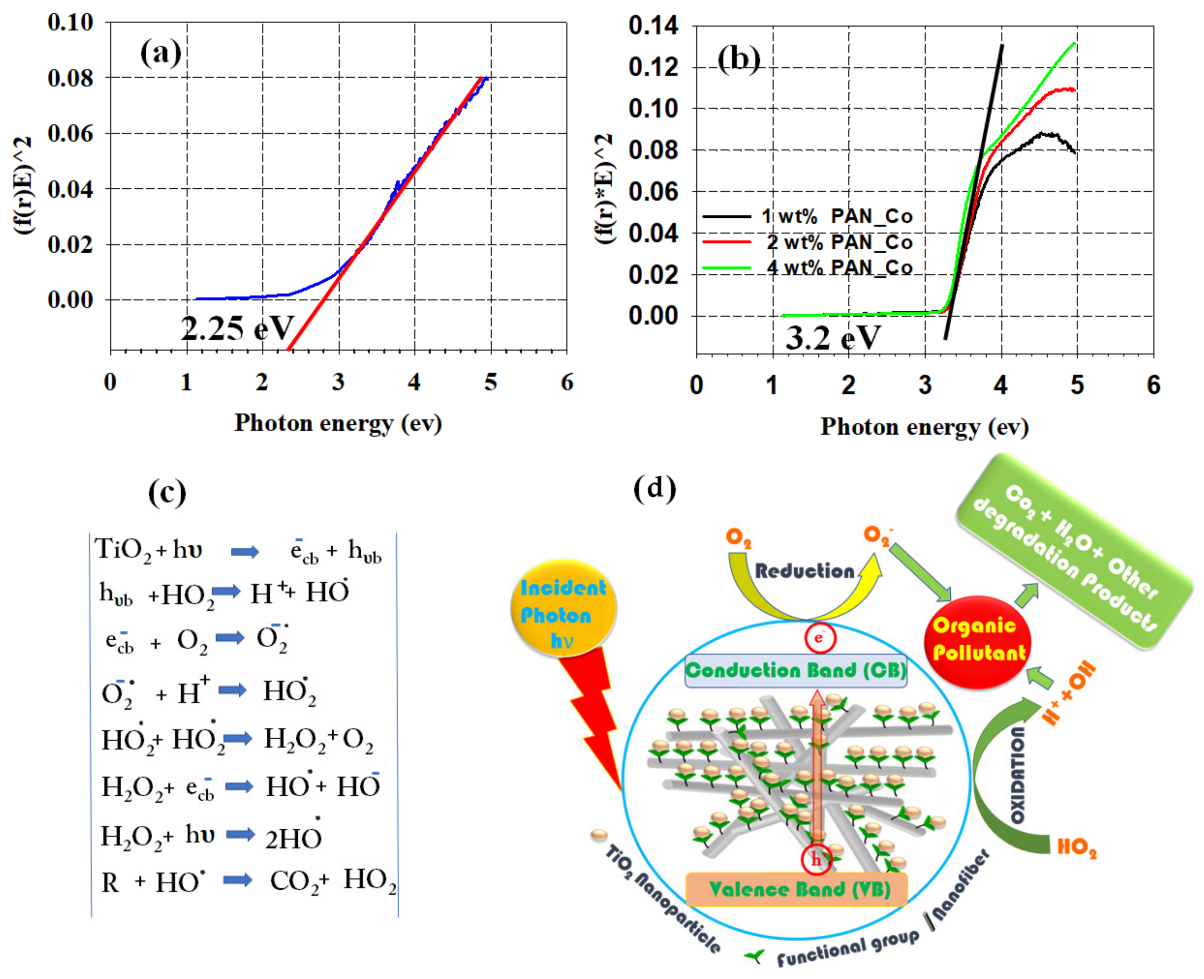
T-plots of the energy band gap (**a**) PAN_Coa, (**b**) PAN_Co, (**c**) reactions during photodegradation of the pollutant and (**d**) schematic of the photocatalysis process. The wt% shows the content of TiO_2_ NPs in the PAN_Co NFs membrane.

The comparison of PAN_Coa with PAN_ Co NFs membranes at constant dosage (60 mg), dye concentration (20 ppm) and loading of TiO_2_ NPs (4 wt%) is shown in Figure 8b,f and Figure 9c,f. From the figures, it can be seen that PAN_Coa NFs membrane outperformed PAN_Co NFs membrane in terms of photocatalytic activity. PAN_Coa and PAN_Co NFs membranes had a degradation efficiency of 92.68 and 41.64%, respectively (Figure 8d,f and Figure 9d,f). Rate constant for TiO_2_ coated DETA-f-PAN NFs membrane with variation in wt.% of TiO_2_/PAN membrane was shown in Figure S1. Rate constant for TiO_2_ coated DETA-f-PAN NFs membrane with variation in membrane concen-tration of methyl orange was depicted in Figure S2. However, increasing the amount of TiO_2_ NPs from 1 wt% to 4 wt% in the PAN_ Co showed a slight increase in dye degradation from 19.37 to 41.64% (Figure 9f). The comparison of the above results with the photocatalytic activity of the bare TiO_2_ NPs (using the optimized photocatalyst dosage and dye concentration) showed that the photocatalytic activity of the bare TiO_2_ NPs (49.60%) was comparable only to the PAN_Co NFs membrane (Figures S3 and S4). Therefore, it was concluded that the PAN_Coa NFs membrane not only exhibited better photocatalytic activity than the PAN_Co NFs membrane, but also that of the bare TiO_2_ NPs [55]. The better photocatalytic activity of the PAN_Coa NFs membrane can be attributed to several factors. These factors include the adsorption of methyl orange on the PAN_Coa NFs membrane due to amine functionality [56], the lower band gap energy (~2.25 eV for PAN_Coa and 3.2 eV for PAN_C) (Figure 10), which is due to the interaction of TiO_2_ NPs with the nitrogen of amine in PAN_Coa NFs membrane; and the higher surface roughness of the membrane (Ra 13.14 µm), which promotes the photocatalytic activity by reflecting the photons, resulting in higher photon absorption [57,58].

## 4. Conclusions

In this work, the PAN_P NFs membrane was prepared by electrospinning. The PAN_P NFs membrane was functionalized to the PAN_F NFs membrane. TiO_2_ NPs were anchored on the PAN_F NFs membrane to prepare PAN_Coa. A composite membrane (PAN_Co) was also prepared by embedding TiO_2_ NPs into the NFs. The PAN_P, PAN_F, PAN_Coa and PAN_Co NFs membranes were characterized by standard microscopic, spectroscopic and X-ray techniques prior to their application for the photocatalytic degradation of methyl orange. SEM micrograph showed a smooth morphology for the PAN_P NFs membrane; the morphology remained the same after functionalization (PAN_F). SEM micrograph also showed a dense cloud of TiO_2_ NPs on the surface for PAN_Coa. The incorporation of amine functional group into the chemical structure of PAN was confirmed by ATR-IR spectra. TEM images showed that the particle size of TiO_2_ NPs varied between 18 and 32 nm. The particles tended to be spherical and highly crystalline. The anatase phase of the TiO_2_ NPs was confirmed by the XRD pattern. EDX and XRD confirmed the presence of TiO_2_ NPs in PAN_Coa and PAN_Co NFs membranes. Surface profilometry showed that surface roughness increased with functionalization and coating. The BET analysis showed that all NFs membranes had comparable isotherms and hystereses. According to IUPAC, the isotherms and hystereses were categorized as type IV and H3, corresponding to mesopores and slit pores. The higher photocatalytic activity of PAN_Coa NFs membrane (92%, 20 ppm) compared to PAN_Co NFs membrane (41.64%) and bare TiO_2_ NPs (49.60%) was attributed to the synergy in adsorption, smaller band gap, high surface roughness and surface area. 

## Figures and Tables

**Table 1:** Surface area and porosity of the NFs membranes.

Samples Name (NFs)	Surface Area (m^2^/g)	Pore Volume (cm^3^/g)	Pore Size (A°)	Total Area in Pores (m^2^/g)
PAN	15.94	0.05	128.71	8.16
PAN Co	17.28	0.07	167.81	10.12
PAN-f	15.22	0.06	155.23	8.76
PAN Coa	22.70	0.08	157.37	8.80

## Data Availability

Not applicable.

## Supplementary Materials

The following are available online at https://www.mdpi.com/article/10.3390/membranes11100785/s1, Figure S1: Rate constant for TiO_2_ coated DETA-f-PAN NFs membrane with variation in wt.% of TiO_2_/PAN membrane Keeping the concentration of methyl orange 20 ppm and dose 60 mg, Figure S2: Rate constant for TiO_2_ coated DETA-f-PAN NFs membrane with variation in membrane concentration of methyl orange keeping the dose constant (60 mg), Figure S3. Spectrophotometer spectra of the 20 ppm methyl orange at 0 min and 240 min. TiO_2_ NPs dose was 60 mg, Figure S4. Comparative data of the bare TiO_2_ NPs, PAN_Co and PAN_Coa at 20 ppm methyl orange and 60 mg dose.
