# Supplementary material for: Efficient Photocatalytic Degradation of Organic Pollutant in Wastewater by Electrospun Functionally Modified Polyacrylonitrile Nanofibers Membrane Anchoring TiO2 Nanostructured
Source: Membranes (Basel). 2021 Oct 14;11(10):785. doi: 10.3390/membranes11100785 (PMC8540019; doi:10.3390/membranes11100785)
Supplement: Supplementary file 1 [file membranes-11-00785-s001.zip › membranes-1379237-supplementary.pdf]

membranes

Supplementary Materials

Efficient Photocatalytic Degradation of Organic Pollutant in Wastewater by Electrospun Functionally Modified Polyacrylonitrile Nanofibers Membrane anchoring TiO_2 Nanostructured

Fahad A. AlAbduljabbar [1], Sajjad Haider [2,*], Fekri Abdulraqeb Ahmed Ali [2], Abdulaziz A. Alghyamah [2], Waheed A. Almasry [2], Raj Patel [1] and Iqbal M. Mujtaba [1,*]

[1] Department of Chemical Engineering, Faculty of Engineering & Informatics, University of Bradford, Bradford BD7 1DP, West Yorkshire, UK; thelastfahad@yahoo.com (F.A.A.); R.Patel@bradford.ac.uk (R.P.)

[2] Department of Chemical Engineering, College of Engineering, King Saud University, P.O. Box 800, Riyadh 11421, Saudi Arabia, Saudi Arabia; falhulidy@ksu.edu.sa (F.A.A.A.); aalghyamah@kus.edu.sa (A.A.A.); walmasry@ksu.edu.sa (W.A.A.)

* Correspondence: shaider@ksu.edu.sa; shaider@ksu.edu.sa (S.H.); Iqbal Mujtaba I.M.Mujtaba@bradford.ac.uk (I.M.M)

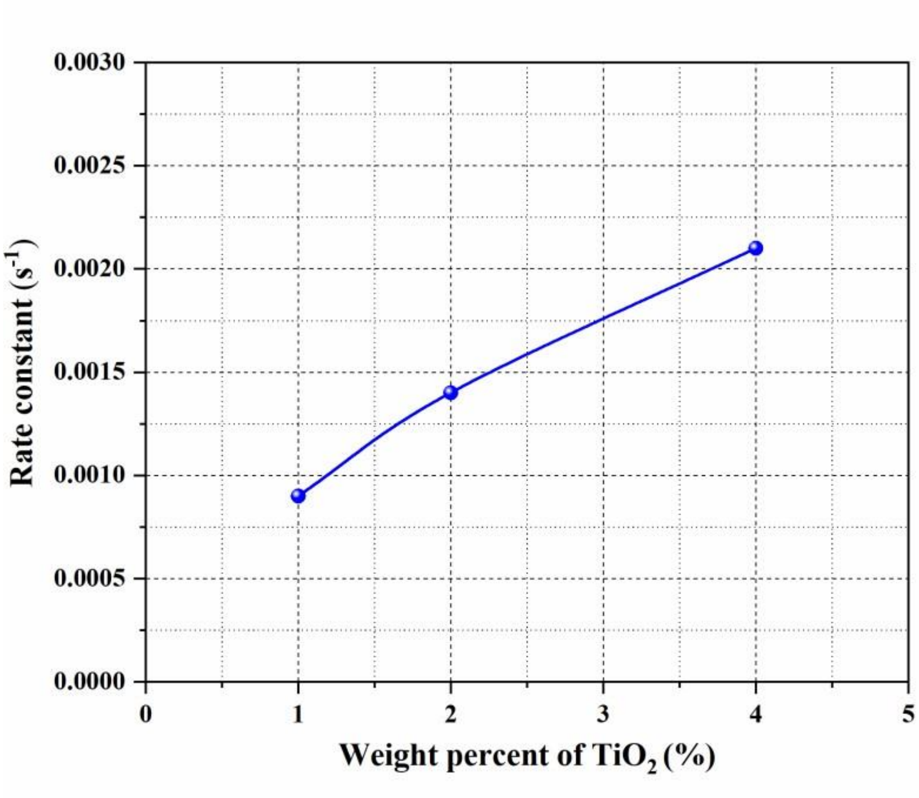

Figure S1. Rate constant for TiO2 coated DETA-f-PAN NFs membrane with variation in wt.% of TiO2/PAN membrane Keeping the concentration of methyl orange 20 ppm and dose 60 mg.

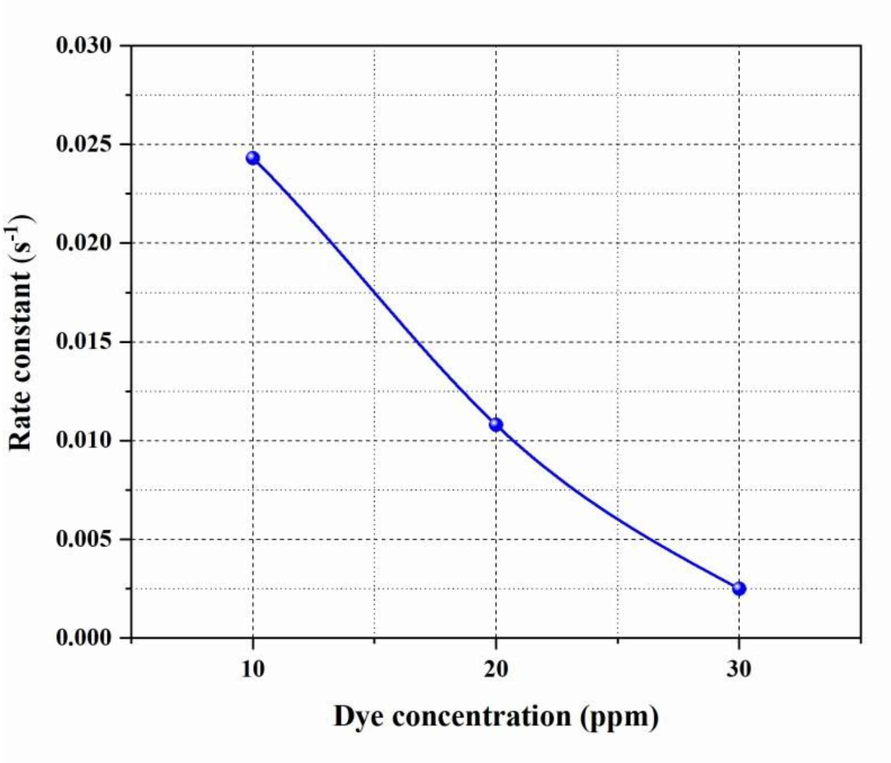

Figure S2. Rate constant for TiO2 coated DETA-f-PAN NFs membrane with variation in membrane concentration of methyl orange keeping the dose constant (60 mg).

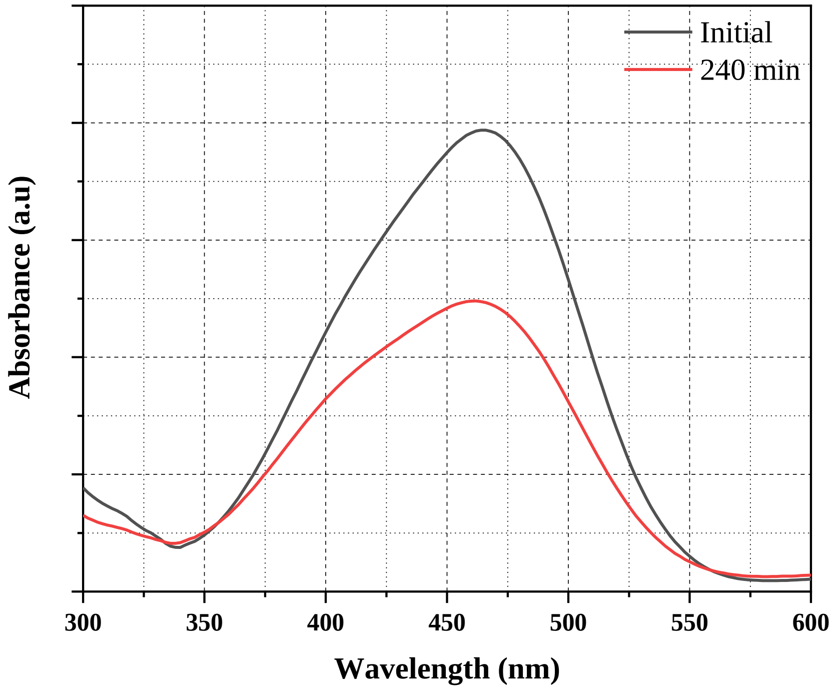

Figure S3. Spectrophotometer spectra of the 20 ppm methyl orange at 0 min and 240 min. TiO2 NPs dose was 60 mg.

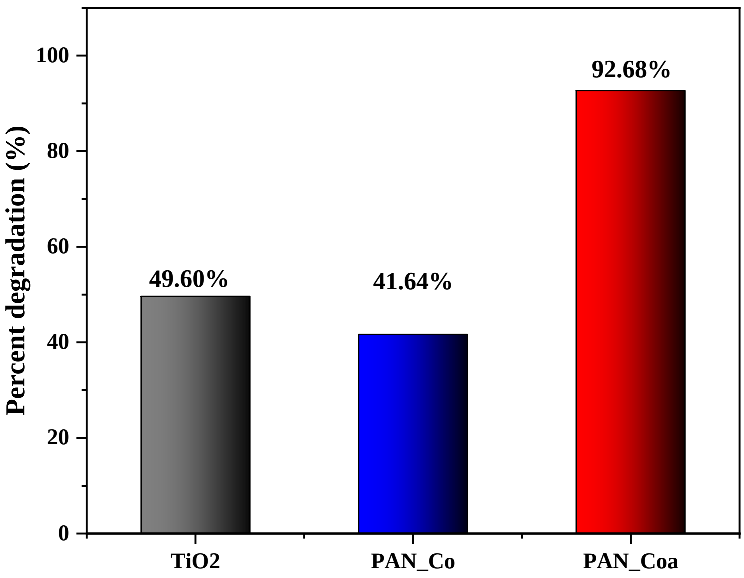

Figure S4. Comparative data of the bare TiO_2 NPs, PAN_Co and PAN_Coa at 20 ppm methyl orange and 60 mg dose.
